# The impact of sulfur mustard on quality of life and mental health in Kurdish survivors in Sweden, thirty years after exposure

**DOI:** 10.1186/s12955-022-02081-y

**Published:** 2022-12-20

**Authors:** Faraidoun Moradi, Fazil Moradi, Ying Li, Anna-Carin Olin, Bledar Daka

**Affiliations:** 1 Occupational and Environmental Medicine, School of Public Health and Community Medicine, Institute of Medicine, University of Gothenburg, Sahlgrenska Academy, Box 414, 405 30 Göteborg, Sweden; 2 Faculty of Humanities, Johannesburg Institute for Advanced Study, University of Johannesburg, Johannesburg, South Africa; 3 School of Public Health and Community Medicine, Institute of Medicine, University of Gothenburg, Sahlgrenska Academy, Göteborg, Sweden; 4 School of Public Health and Community Medicine, Institute of Medicine, Sahlgrenska Academy, University of Gothenburg, Göteborg, Sweden

**Keywords:** Quality of life, Depression, Sulfur mustard, SF-36, MADRS-S, Sweden, Kurdistan

## Abstract

**Background:**

The Iraqi state used chemical warfare agents (CWAs) like sulfur mustard (SM) in al-Anfal genocide in the present-day Kurdistan Region of Iraq. In addition to somatic injuries, exposure to CWAs causes biopsychosocial complications. We investigated the long-term impact of SM exposure on quality of life (QoL) and depression severity in Kurdish survivors resettled in Sweden.

**Methods:**

This is a case-control study, where subjects exposed to SM (n = 18, mean age 51.3 years, 50% women) and sex- and age-matched nonexposed subjects (n = 30, mean age 48.7 years, 47% women) of Kurdish residents in Sweden. Data were collected through in-person interviews based on the RAND 36-item Short Form Health Survey to assess QoL and the Montgomery-Åsberg Depression Rating Scale-self assessment (MADRS-S) to investigate the presence and the gravity of depressive symptoms.

**Results:**

The SM-exposed group had a significantly lower QoL than the nonexposed group (*p* < 0.001). Also, the overall mean MADRS-S scores among the SM-exposed group, corresponding to moderate depression, were higher than the scores of the nonexposed (22 points (p) vs. 9 p, *p* < .001). Overall, the participants within the exposed group reported worse mental than physical well-being 36p and 32p, respectively. Within the SM-exposed group, there was no gendered-related difference neither in terms of depression severity nor for QoL, but the groups were small.

**Conclusion:**

Individuals exposed to SM had worse QoL and a higher level of depressive severity compared with nonexposed individuals three decades after exposure, indicating the importance of increased clinician knowledge, guidelines, and an approach to assess and respond to the exposed groups’ biopsychosocial needs. These findings indicate that those exposed to SM might need early identification of mental illnesses and more support to promote QoL.

## Background

Chemical warfare agents (CWAs) have been used during World War I and II, the Holocaust, and sporadically in many international, regional, and civil wars despite global legal prohibition [1–3]. Terrorist groups have used CWAs to incite fear and instability among civilian populations [1, 3, 4]. Industrial chemical accidents and environmental disasters also contribute to chemical exposure [5, 6]. Sulfur mustard (SM) is one of the most widely used CWAs. It was extensively employed in the Iraq–Iran war (1980–88) [3]. During al-Anfal genocide (1987–1991), the Iraqi state used SM and sarin against Kurdish political organizations, armed forces, and civilian populations in the present-day Kurdistan Region of Iraq (KRI) [7]. The chemical bombardment of Halabja city on March 16, 1988, left thousands dead and injured for life and remains unprecedented in the post-World War II era [8]. The survivors in Halabja were exposed to different CWAs, e.g., SM and sarin [9]. Thus, SM has a higher morbidity rate than nerve agents and can cause late-onset biopsychosocial complications [10]. SM-related health effects develop gradually—for example, respiratory symptoms and psychological disorders such as mood and depressive disorders, prolonged post-traumatic stress disorder, and chemical contamination anxiety impact all aspects of survivors’ lives [11]. Moreover, SM-exposed people’s health deteriorates over time, resulting in greater healthcare service use [3, 9], possibly impacting survivors’ quality of life (QoL) [12].

Ongoing wars in the Middle East and international conflicts have forced hundreds of Kurdish, Syrian, and Iranian CWA survivors to migrate and resettle abroad (e.g., Sweden). There is an urgent need for more knowledge to improve medical care and treatment in preparation for CWA exposure and survivors. Previous studies reported that female survivors of CWA exposure experienced more pronounced psychosocial adverse effects than males [9, 13].

Survivors at risk for severe chronic disease and impaired QoL in their later years need follow-up healthcare. There is limited knowledge regarding the mental health and QoL of persons exposed to SM in western countries. However, the long-term impacts of CWA, e.g., SM on QoL and mental health in Iranian veterans, are highlighted in multiple studies [11, 14, 15].

This study aimed to assess, evaluate, and compare the QoL and mental health condition of Kurdish survivors resettled in Sweden (exposed) compared to a nonexposed group of Kurdish residents in Sweden (i.e., controls).

Scholars have used the Short Form Health Survey 36 (SF-36) questionnaire to obtain an overall QoL score and use public health-related evidence to influence policymakers [16]. Similarly, clinicians employ the self-rated Montgomery-Åsberg Depression Rating Scale (MADRS-S) as a supplementary assessment to monitor depression severity [17].

We hypothesized that survivors in the exposed group would report impaired QoL and poor mental health compared to the matched nonexposed group.

## Methods

### Study design and participants

This case-control study involved two groups of Kurdish-Swedish citizens residing in Sweden. Participants provided informed written consent before inclusion. This study adhered to the principles of the Declaration of Helsinki, and the Regional Ethical Review Committee in Gothenburg, Sweden, approved it (599-17).

To recruit participants, we performed a purposive recruitment approach that supported diversity (in sex and age). We used different strategies to recruit potential participants: posters, social media announcements, local radio, and word-of-mouth. Recruiting subjects for the study has been challenging because survivors who continue to suffer from the effects after exposure feel taboo and do not want to talk about their daily uneasiness.

For people in the exposed group, the eligibility criteria included: (1) they came from SM-attacked areas in the KRI and survived the chemical attacks of 1987 and 1988—with a particular interest in survivors from Halabja; (2) they developed physical symptoms during the chemical bombardment (to affirm exposure) and (3) they were aged between 30 and 80.

We excluded individuals who were unavailable for a conversation longer than 30 min and people exposed to other significant trauma (e.g., prison or torture) during al-Anfal genocide. Regarding the nonexposed group, inclusion criteria stipulated that participants were originally from the KRI, had no history of SM exposure, and were aged between 30 and 80.

For the control group, we selected the non SM-exposed first-generation Kurdish-Swedish citizens in Sweden rather than the general Swedish population since their similar sociodemographic factors as the exposed group. The study protocol was planned to match for age and sex; 1:2 exposed to nonexposed participants. Nevertheless, two participants dropped out of the nonexposed group by not giving written consent, and four did not respond to a telephone reminder to fill in questionnaires.

### Exposure assessment

We determined the exposure history based on participants’ lived experiences, memories, and testimonies. No objective medical method was used to confirm SM exposure. However, the development of physical symptoms during the chemical bombardment is often used to affirm exposure. The primary exposure was defined as direct contact with or inhalation of SM owing to the explosion of nearby bombs. Secondary exposure is indirect contact through inhaling polluted air or touching contaminated bodies.

### Measures

The Swedish version 1.0 of the standardized RAND SF-36 was used to determine QoL [18]. This tool has previously been validated and is widely used to measure QoL in patients and the general public. The RAND SF-36 variables were summarized in an overall score of optimal physical well-being, represented by the Physical Component Summary (PCS), which included physical functioning, role limitations owing to physical health, bodily pain, and general health [19]. Mental well-being was expressed through the Mental Component Summary (MCS), which included vitality, social functioning, role limitations owing to emotional problems, and mental health [19].

We used the Swedish version of the MADRS-S to quantify depression severity [17, 20]. The MADRS-S is often used in clinical practice in Scandinavia and comprises nine questions [17, 20].

We used manual guidelines to calculate and interpret the RAND SF-36 survey [21], and compiled respondents’ responses into pre-coded items before coding them into final item values. Finally, each absolute item value was transformed and converted to scores ranging from 0–100 using a unique formula: 0 indicates the poorest QoL, and 100 represents the optimal QoL. The combined PCS and MCS were estimated for each participant group using standard (U.S.-derived) scoring algorithms in User Manual 1994 [19] in three steps: first, scale standardization; second, scale scores combination; and finally, summary scores transformation. The MADRS-S items were calculated and compiled; with each item yielding a score of 0–6 points, the overall score ranges from 0 to 54. Higher scores indicate severe levels of depressive symptoms [17].

### Data collection

The data for the RAND SF-36 and MADRS-S questionnaires in the exposed group, including their sociodemographic data and the nature of their SM exposure, were collected via in-person interviews with survivors in Sweden between January 2018 and August 2018 (Table 1). Interviews were held at a place of the participant’s choosing. Data concerning the RAND SF-36, MADRS-S, and the demographic variables of the nonexposed group were mainly self-reported. We completed the inadequate questionnaires later via telephone calls.

**Table 1:** Participants’ demographic, socioeconomic and route of sulfur mustard exposure characteristics (n = 48)

Variable	Nonexposed (n = 30)	Exposed (n = 18)	P*
Sex, number of Women (%)	14 (47)	9 (50)	0.53
Age (years), mean (SD) Median (Min–Max)	48.7 (10.3) 51.5 (32–67)	51.3 (8.2) 51.5 (30–66)	0.37
Body mass index (kg/m^2^), mean (SD) Median (Min–Max)	27.4 (3.9) 26.5 (22.8–39)	27.2 (3.1) 25.9 (23–33)	0.64
Educational achievement, n (%)			0.02
≤ Primary	1 (3)	6 (33)	
High school	12 (40)	7 (39)	
≥ University	17 (57)	5 (28)	
Employment status, n (%)			0.01
Unemployed	4 (13)	9 (50)	
Marital status, n (%)			0.58
Married	22 (73)	12 (67)	
Single	1 (3)	2 (11)	
Divorced	7 (23)	4 (22)	
Type of exposure, n (%)			
Primary		11 (61)	
Secondary		7 (39)	

*n* Number of participants, *SD* Standard deviation

For categorical variables, n (%) is presented; for continuous variables Mean (SD)/ Median (Min—Max) is presented  . P* are based on independent T-tests or Fisher exact test, depending on the type of the variables

### Statistical analysis

The differences in the background variables, including, sex, age, BMI, education achievement, employment, and marital status, were tested using an independent T-test or Fisher’s exact, depending on the type of the variables. As a result, education achievement and employment status showed significant differences between groups; therefore, they will be considered in the later analysis.

As the variables of the RAND SF-36 and MADRS-S scores were not normally distributed, Mann–Whitney U-test was used to test differences between the exposed and nonexposed groups.

For the summary scores, i.e., PCS, MCS, and overall mean of MADRS-S, their distributions were relatively symmetric and suitable for regression analysis.

We conduct a direct acyclic graph to find out the mechanic pathways between exposure, education, employment status, and outcome scores. Although education may have a direct effect on the outcome theoretically, but there was no significant association between education and outcomes in our data with *p* > 0.3. Therefore, the mediation pathway includes both education and employment. We conduct mediation analysis to separate exposure's direct and indirect effect on PCS, MCS, and overall mean of MADRS-S using package "mediation" in R. The statistical analyses were performed using IBM SPSS 27.0 software (SPSS, Chicago, IL) and R 4.0.2.

## Results

There were 18 participants in the exposed group (n = 18, mean age = 51.3 ± 8.2 years, 50% women) and 30 in the nonexposed group (mean age = 48.7 ± 10.3 years, 47% women). Details of participants’ demographic, socioeconomic, and nature of SM exposure variables are presented in Table 1. There were no significant differences concerning sex, age, body mass index, or marital status between groups. Higher unemployment and a low education level were observed in the exposed than in the nonexposed group. Furthermore, 61% (11 subjects) in the exposed group experienced primary SM exposure.

### Quality of life

Table 2 shows the descriptive statistics in QoL perception in both groups. The exposed group had significantly lower outcomes than the nonexposed group for all eight RAND SF-36 variables and the two combined PCS (mean of 36.2 in the exposed vs. 45.7 in control, *p* = 0.002) and MCS (mean of 32.2 in the exposed and 45.5 in control, *p* = 0.002). The most profound differences were for vitality, social function, bodily pain, and general perception of health.

**Table 2:** Descriptive statistics on RAND Short Form Health Survey 36-items and the combined MCS/PCS in the exposed and nonexposed groups

Variable	Nonexposed (n = 30)	Exposed (n = 18)	P*
	Mean (SD) Median (Min–Max)	Mean (SD) Median (Min–Max)	
Physical function	77.5 (17.3) 77.5 (25–100)	60.0 (22.9) 57.5 (30–95)	0.011
Role physical	65.0 (37.5) 75.0 (0–100)	34.7 (45.5) 0.0 (0–100)	0.023
Role emotional	66.7 (40.1) 83.3 (0–100)	35.2 (47.8) 0.0 (0–100)	0.031
Social function	72.1 (19.3) 62.5 (50–100)	43.1 (28.2) 37.5 (0–100)	< 0.001
Bodily pain	58.6 (24.1) 62.0 (0–100)	33.6 (25.4) 26.5 (0–90)	0.001
Mental health	62.0 (21.9) 66.0 (0–92)	39.5 (25.1) 34.0 (4–100)	0.002
Vitality	53.8 (19.6) 55.0 (15–85)	29.2 (24.7) 25.0 (0–100)	< 0.001
General health	61.0 (19.7) 67.0 (17–100)	26.5 (26.3) 15.0 (0–100)	< 0.001
Physical component summary	45.8 (7.9) 44.9 (27–58)	36.2(9.6) 41.4 (21–56)	0.002
Mental component summary	44.5 (11.6) 44.8 (16–70)	32.2 (13.4) 28.4(11–63)	0.002

*PCS* Physical component summary, *MCS* Mental component summary, *n* Number of participants, *SD* Standard deviation

A higher score indicates a better quality of life. *P is based on independent-Samples Mann–Whitney U Test

### Depression

Table 3 shows results from MADRS-S outcomes and participants’ perceptions of their depressive symptoms’ severity. Expectedly, the exposed group scored significantly higher on all nine of the MADRS-S variables and overall mean scores than the nonexposed group (*p* < 0.001). The nonexposed group’s overall mean MADRS-S score was 9.1 with SD 7 p, which did not meet the criterion for depression [20]. The exposed had an overall mean MADRS-S score of 22.9 with SD 12.6 p, which is within the reference range for moderate depressive symptoms [20]. The most significant differences were found for the variables suicidal thoughts, lassitude, inability to feel, pessimistic thoughts, reduced appetite, sleep, and reported sadness compared to the nonexposed group.

**Table 3:** Descriptive statistics on Montgomery-Åsberg Depression Rating Scale (MADRS-S) variables and overall mean scores

Variable	Nonexposed (n = 30)	Exposed (n = 18)	P*
	Mean (SD) Median (Min–Max)	Mean (SD) Median (Min–Max)	
Reported sadness	0.8 (1.1) 0.0 (0–4)	2.9 (1.8) 3.5 (0–6)	< 0.001
Inner tension	2.3 (1.5) 2.0 (0–6)	3.2 (1.7) 4.0 (0–6)	0.036
Reduced sleep	1.8 (1.8) 2.0 (0–6)	3.4 (2.2) 3.5 (0–6)	0.012
Reduced appetite	0.5 (0.8) 0.0 (0–2)	1.6 (1.5) 2.0 (0–6)	0.004
Concentration difficulties	1.4 (1.2) 2.0 (0–4)	2.9 (1.8) 3.5 (0–6)	0.003
Lassitude	0.5 (0.8) 0.0 (0–2)	2.3 (1.8) 2.0 (0–5)	< 0.001
Inability to feel	0.6 (1.0) 0.0 (0–3)	2.4 (1.9) 2.5 (0–6)	0.001
Pessimistic thoughts	0.8 (1.4) 0.0 (0–6)	2.2 (1.8) 2.0 (0–5)	0.002
Suicidal thoughts	0.4 (0.8) 0.0 (0–2)	1.9 (1.9) 2.0 (0–5)	0.002
Overall mean MADRS-S	9.1 (7.0) 8.0 (0–28)	22.9 (12.6) 27.5 (0–41)	< 0.001

*PCS* Physical component summary, *MCS* Mental component summary, *n* Number of participants, *SD* Standard deviation

A higher score indicates a better quality of life. P* are based on independent-Samples Mann–Whitney U test

### Mediation analysis

**Fig. 1:**
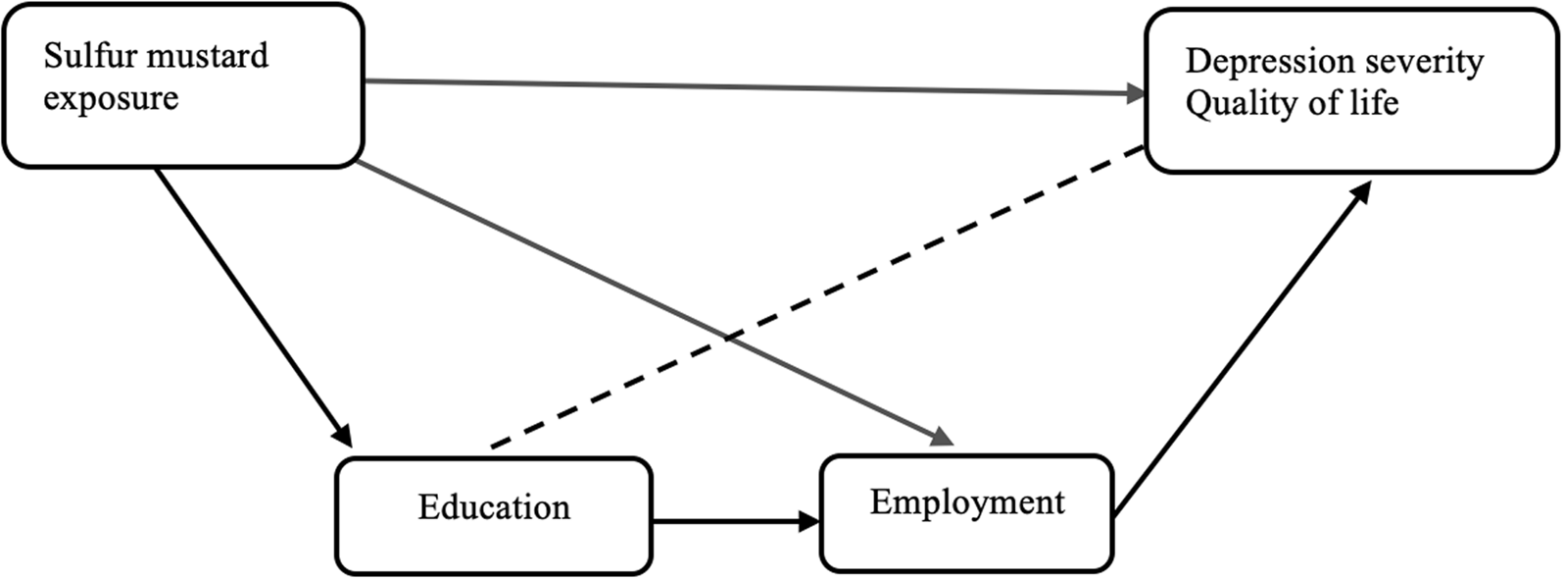
Directed acyclic graph showing the mediators in the association between SM exposure and outcomes

Table 4 reveals that after adjustments, the average mean differences between groups were − 11.9 with 95% CI(− 19.75, − 4.17) on MCS, − 9.11 with 95% CI (− 14.56, − 4.52) on PCS, and 13.40 with 95% CI(5.15, 19.37) on MARDS-S. The proportion of the total effect of exposure mediated by employment pathway was 18% on MCS, 13% PCS, and 21% on MADRS-S. Figure 1 illustrates the mechanic pathways between exposure, education, employment status, and outcomes.

**Table 4:** Total natural direct and natural indirect effects of exposure on MCS, PCS, and overall mean MADRS, with mediation through education and employment

		Coefficients	95% CI of coefficients		*p*
MCS	Natural indirect effect	− 2.22	− 7.21	0.64	0.16
	Natural direct effect	− 9.71	− 19.55	− 0.82	0.04
	Total effect	− 11.93	− 19.75	− 4.17	< 0.001
	Proportion mediated	18%	− 5%	86%	0.16
PCS	Natural indirect effect	− 1.24	− 4.56	0.60	0.30
	Natural direct effect	− 7.87	− 13.42	− 2.49	< 0.001
	Total effect	− 9.11	− 14.56	− 4.52	< 0.001
	Proportion mediated	13%	− 7%	58%	0.30
MADRS-S	Natural indirect effect	3.14	0.05	7.40	0.04
	Natural direct effect	10.26	2.39	17.20	< 0.001
	Total effect	13.40	5.15	19.37	< 0.001
	Proportion mediated	21%	0%	65%	0.04

*PCS* Physical component summary, *MCS* Mental component summary, *MADRS-S* Montgomery-Åsberg Depression Rating Scale

### Gendered health condition

The knowledge regarding the sex-related impacts of CWA exposure is scarce; thus, the existing data indicate long-term biopsychosocial gender differences, where female survivors are more affected than male survivors [13, 22]. In the current study, no significant gender-related differences were found for outcomes of the RAND SF-36 variables (Table 5) and the MADRS-S (Table 6). However, the female survivor’s score for the overall mean MADRS-S was lower than the exposed males, and the number of participants was low.

**Table 5:** Montgomery-Åsberg Depression Rating Scale (MADRS-S) variables and overall mean and median scores by sex within the exposed group (50% women)

Variable	Men (n = 9)	Women (n = 9)	P^*^
	Mean (SD) Median (Min–Max)	Mean (SD) Median (Min–Max)	
Reported sadness	3.0 (1.4) 4.0 (0–4)	2.9 (2.3) 2.0(0–6)	0.863
Inner tension	3.0 (2.0) 4.0 (0–6)	3.4 (1.5) 4.0 (2–6)	0.730
Reduced sleep	3.9 (2.3) 4.0 (0–6)	2.9 (2.1) 3.0 (0–6)	0.340
Reduced appetite	1.9 (1.1) 2.0 (0–4)	1.3 (1.9) 1.0 (0–6)	0.161
Concentration difficulties	2.9 (1.7) 4.0 (0–4)	2.9 (2.0) 3.0 (0–6)	1.000
Lassitude	1.9 (1.6) 2.0 (0–4)	2.7 (1.9) 4.0 (0–5)	0.340
Inability to feel	1.6 (1.7) 1.0 (0–4)	3.2 (1.8) 3.0 (0–6)	0.094
Pessimistic thoughts	2.0 (1.7) 2.0 (0–4)	2.4 (1.9) 3.0 (0–5)	0.666
Suicidal thoughts	1.3 (1.5) 1.0 (0–4)	2.6 (2.1) 4.0 (0–5)	0.222
Overall mean MADRS-S	21.4 (12.2) 27.0 (0–36)	24.3 (13.6) 31.0 (5–41)	0.489

No depression, 1–12; Mild depression, 13–19; Moderate depression, 20–34; Severe depression, > 34. Higher scores indicate poor condition

*n* Number, *SD* Standard deviation

P* are based on independent-Samples Mann–Whitney U Test. Significance was set at *p* < 0.05

**Table 6:** RAND 36-item Short Form Health Survey variables scores by sex within the exposed group (50% women)

Variable	Men (n = 9)	Women (n = 9)	P*
	Mean (SD) Median (Min–Max)	Mean (SD) Median (Min–Max)	
Physical function	63.9 (20.1) 60.0 (35–95)	56·1 (25·5) 45.0 (30–95)	0.436
Role physical	44.·4 (48.1) 25.0 (0–100)	25.0 (43.3) 0.0 (0–100)	0.436
Role emotional	48.1 (50.3) 33.3 (0–100)	22.2 (44.1) 0.0 (0–100)	0.297
Social function	51.4 (26.1) 50. 0 (25–100)	34.7 (29.2) 25.0 (0–88)	0.113
Bodily pain	39.2 (29.8) 31.0 (0–90)	28.0 (20.3) 22.0 (0–62)	0.489
Mental health	40.4 (26.2) 28.0 (20–100)	38.7 (25.6) 44.0 (4–92)	0.730
Vitality	36.1 (27.1) 25.0 (10–100)	22.2 (21.2) 20.0 (0–70)	0.190
General health	29.3 (31.5) 25.0 (0–100)	23.8 (21.6) 15.0 (5–72)	1.000
Physical component summary	34.2 (9.5) 29.4 (21–50)	38.2 (9.5) 34.1 (28–55)	0.258
Mental Component Summary	29.5 (13.6) 27.6 (11–57)	34.9 (13.5) 29.1 (22–63)	0.436

*n* Number of participants, *SD* Standard deviation

P* are based on independent-Samples Mann–Whitney U Test

## Discussion

This study found that SM-exposed survivors had significantly worse and more impaired QoL and reported a high level of moderate depressive symptoms compared to nonexposed controls three decades after the exposure.

Participants in the exposed group reported significantly lower scores in each of the eight RAND SF-36 variables and the two combined physical and mental well-being scores compared with the nonexposed group, which indicates an obvious, worse QoL.

The most pronounced differences regarding QoL variables were social function, bodily pain, vitality, and general perception of health. Overall, the survivors had worse mean scores for mental than physical well-being, possibly because the mental symptoms stemming from SM exposure were challenging to identify and subsequently hard to treat—regardless of healthcare access. Conversely, physical well-being depends on healthcare access and can vary according to local resources. It may be easier for exposed participants to receive treatment for physical symptoms as opposed to psychological ones.

The apparent discrepancy in reported impaired QoL and worse mental well-being in the exposed group compared with the nonexposed may be attributed to the fact that participants in the exposed group were exposed to SM (Table 1). Notably, a previous study showed that more significant organ damage caused more acute and long-term illness among exposed persons than the controls [14]. Thus, direct SM exposure experienced by the survivor’s group may have led to harsher physical outcomes.

The impaired mental illness observed are some apparent factors behind reduced workability and unemployment, previously reported among SM-exposed survivors, that may contribute to impaired QoL [2, 9]. This might be attributed to post-exposure health conditions (e.g., respiratory symptoms and fatigue) [2, 9]. Furthermore, the associations between chronic health conditions, low QoL, and unemployment are well known among patient groups with no CWAs exposure [23]. Another notable pattern that emerged is the lower level of education among survivors. Previous research indicates that education is independently linked with employment status, and studies have reported an association between poor health and low education [24]. It might be explained by the fact that SM-exposed survivors have difficulties following education due to post-exposure symptoms, e.g., reduced concentration and mental illness. Despite the welfare system in Sweden, unemployment results in low income, a contributing factor to poor mental health and low QoL and vice versa [25].

Mental health is an essential component of good QoL. SM exposure results in late-onset psychological issues: prolonged post-traumatic stress syndrome, depression, and chemical contamination anxiety [3, 9]. Predictably, participants in the SM-exposed group reported predominantly higher scores for symptoms of depression than the nonexposed group (Table 3). Our MADRS-S findings highlighted more severe depressive symptoms among survivors, with mean MADRS-S scores of 22.9 points (Table 3). These results were in line with the outcomes of RAND SF-36 scores, in which survivors rated their combined MCS lower than their PCS (Table 2). Additionally, the most pronounced differences were that survivors experienced a four-fold higher risk for suicidal thoughts, lassitude, and inability to feel, a three-fold higher risk for pessimistic thoughts, reduced appetite, and reported sadness, and a two-fold higher risk for reduced sleep (Table 3). These symptoms are understood as post-exposure complications and have been reported in SM-exposed survivors in Iran and Kurdistan-Iraq and even in World War I veteran survivors [2, 3, 9].

The pathophysiological aspects of neuropsychiatric illnesses and the inevitable social effects are not thoroughly studied in SM-exposed survivors. However, brain impacts structurally and functionally were documented after exposure to SM or its analog and in veterans with War-related PTSD [26–28]. A recent study has revealed that the prevalence of post-exposure reduced sleep is associated with a lower level of serum melatonin (a hormone playing an essential role in promoting sleep) in SM-exposed patients [28]. Furthermore, brain damage has been revealed in mice exposed to SM analog vesicant [26]. Fatigue and poor vitality are linked to SM post-exposure respiratory symptoms and reduced sleep affecting many aspects of an exposed individual’s life. Also, functional magnetic resonance imaging in veterans with Gulf War-related PTSD showed a reduced hippocampus volume compared with the healthy control group [27]. Several studies, have reported that the mental illnesses and symptoms in SM-exposed survivors are associated with damage to the nervous system [2, 3, 9]. The brain “abnormalities” in SM-exposed people might impact their cognitive ability in the long-term [26–28]. This could explain the relationship between exposure to SM and poor mental health conditions, low QoL, and low education level despite the extreme complexity of the interrelation between these factors. But, despite these nuances, multiple studies have indicated that exposure to CWAs—even the fear of being exposed to them—leads to a chronically impaired health status and low QoL [3, 29]. These factors were strongly associated with low academic performance and unemployment [24], which coincides with our current findings.

However, the survivors in the exposed group had consistent access to public healthcare services and an established welfare system. Despite this, survivors in Sweden occupy marginal social, economic, and cultural positions and have a poor state of health. A study showed that foreign-born people rated their health poorer than those born in Sweden [30]. This study demonstrates that SM exposure is associated with enduring long-term poor mental well-being and impaired QoL. Consequently, the results indicate that the healthcare service systems might need new adapted approaches and increased knowledge for the early identification of mental illness among SM-exposed survivors.

The current study showed slightly non-significant gender differences in survivors’ perception of QoL and mental illness (Tables 5 and 6), which may be explained by the small sample size. Another explanation might be the more independent sex, economic, and social possibilities and equalities in Swedish society. Thus, survivors have enjoyed an equal individual mode of existence and access to healthcare services.

A new study showed that having a good relationship with a partner was associated with perceived good health [31]. In our previous study among survivors in Halabja, we revealed that SM exposure survivors suffered from poor marital relations and female survivors reported difficulties getting married which might contributed to impaired QoL [13].

Furthermore, survivors were characterized by a significantly higher degree of unemployment and lower education than the nonexposed group, which was consistent with previous studies [9, 15, 32]. The education and unemployment pathways partly mediated the outcome differences between exposed and nonexposed groups (Fig. 1). Nevertheless, exposure to SM has the dominant direct impact on mental health and QoL in terms of both physical and mental well-being and the gravity of depression (Table 4).

Remarkably, the exposed group had lower QoL and combined MCS compared to the studies conducted among Iranian male veteran survivors living in Iran [14, 15]. Furthermore, our results showed that SM-exposed survivors in Sweden reported higher(better) scores in terms of PCS variables; physical function, role physical, bodily pain, and general health than the Iranian male survivors of CWA. However, there were slight differences in MCS variables; social function, role emotional, vitality, and mental health [15]. This comparison has limitations, including the number and sex of the participants. However, the male survivors in the exposed group in our study scored about seven points lower on mean MCS than the Iranian male survivors[14]. This can be attributed to the lack of experience, clinician knowledge, and guidelines for treating exposed persons in the Swedish healthcare system or other barriers male participants may have encountered, such as language barriers and social exclusion. Another explanation might be that the Iranian war survivors were consistently provided the highest quality of long-term care services and were treated and hospitalized after SM exposure [3, 33].

Nevertheless, the apparent differences in impaired QoL and high depressive symptoms in the SM-exposed compared with the nonexposed group highlights the reinforced possible association between SM exposure and higher unemployment levels, low education levels, low QoL, and long-term poor mental well-being among survivors. Our results were confirmed by previous studies that determined CWA survivors experienced poor mental well-being and impaired QoL [3, 11, 12, 14, 15, 33].

## Limitations

This study had some limitations. First, low participant numbers resulted in minor frequencies, which might limit generalizability to larger populations. However, the interesting results of this paper play an essential role in giving a voice to the everyday challenges and difficulties of this almost-forgotten group of patients. A second limitation was that our results were based on lived experiences, memories, and reported symptoms that were part of participants’ narratives. However, having a laboratoristic investigation of being SM-exposed is impossible, and access to medical journals is not possible due to the lack of medical journals. The survivors in Halabja were exposed to different CWAs, e.g., SM and sarin [9], and therefore, it is difficult to rule out overlapping and synergistic effects on the participants. Thus, unlike sarin, SM has lower mortality but can cause late-onset biopsychosocial complications [10]. In other studies, the SM-exposed participants have been recruited from a database managed by the authorities [9, 32]. Furthermore, it is impossible to comment on the causality between exposure to SM and this paper's outcomes due to the study's observational nature. Finally, direct or indirect experiences of other traumatic violence may have influenced the outcomes of MADRS-S and RAND SF-36, although the results were consistent with other studies [3, 11, 15].

## Conclusions

Exposure to SM has obvious long-term severe impacts on survivors’ QoL and mental health. We suggest that in addition to the medical focus on this group of patients, one goal within a healthcare system should be to optimize survivors QoL and psychosocial well-being. These findings indicate that SM survivors may benefit from the early identification of a mental illness and more support to promote QoL.

## Data Availability

The datasets used during the current study are available from the corresponding author upon reasonable request.
